# *In vivo* and *in vitro* postovulatory aging: when time works against oocyte quality?

**DOI:** 10.1007/s10815-022-02418-y

**Published:** 2022-03-21

**Authors:** Valentina Di Nisio, Sevastiani Antonouli, Pauliina Damdimopoulou, Andres Salumets, Sandra Cecconi, On behalf of SIERR

**Affiliations:** 1 Division of Obstetrics and Gynecology, Department of Clinical Science, Intervention and Technology, Karolinska Institutet and Karolinska University Hospital, 14186 Huddinge, Stockholm Sweden; 2 Department of Life, Health and Environmental Sciences, University of L’Aquila, Via Vetoio, 67100 L’Aquila, Italy; 3 Department of Obstetrics and Gynaecology, Institute of Clinical Medicine, University of Tartu, 50406 Tartu, Estonia; 4 Competence Centre On Health Technologies, 50411 Tartu, Estonia

**Keywords:** Postovulatory aging, Oocyte, Oxidative stress, Morphological alteration, Assisted reproductive technology, Antiaging chemicals

## Abstract

In mammalian species an optimal fertilization window during which successful fertilization occurs. In the majority of mammals estrus marks ovulation time and coincident with mating, thereby allowing the synchronized meeting in the fallopian tubes, between freshly ejaculated sperm and freshly ovulated oocytes. Conversely, women do not show natural visual signs of ovulation such that fertilization can occur hours later involving an aged oocyte and freshly ejaculated spermatozoa. During this time, the oocyte undergoes a rapid degradation known as “postovulatory aging” (POA). POA may become particularly important in the human-assisted reproductive technologies, as the fertilization of retrieved mature oocytes can be delayed due to increased laboratory workload or because of unforeseeable circumstances, like the delayed availability of semen samples. This paper is an updated review of the consequences of POA, either *in vivo* or *in vitro*, on oocyte quality with particular attention to modifications caused by POA on oocyte nuclear, cytoplasmic, genomic, and epigenetic maturation, and embryo development.

## Introduction

Mature oocytes arrested at meiotic metaphase II (MII) can be successfully fertilized if the insemination occurs in a restricted time window after ovulation, within 12 h for rodents and 24 h for monkeys and humans. Otherwise, oocytes undergo a time-dependent process of degradation referred to as “postovulatory aging” (POA). In comparison with oocytes analyzed soon after ovulation, these aged oocytes evidence several morphological, molecular, genomic, and epigenetic anomalies [1–6] that can even result in apoptosis [1, 2, 7]. In humans, oocytes retrieved during the assisted reproduction technology (ART) procedures can be subjected to extended periods of culture prior to fertilization, thus reducing the viability of the resulting embryos [3]. Both *in vivo* and POA have been associated with reduced fertilization rates, poor embryo quality, implantation failure, and abnormalities in the offspring [3].

In contrast with the physiological fertilization [8, 9], the common effects of POA in oocytes are the degeneration of polar body (PB) I, the incomplete extrusion of PBII associated with disruption of meiotic spindle [10], increased perivitelline space and partial cortical granules (CGs) exocytosis [11]. Other morphological modifications such as shrinkage, membrane blebbing, cytoplasmic fragmentation and granulation, and degeneration [9, 12] are mainly caused by increased reactive oxygen species (ROS) production.

**Fig. 1:**
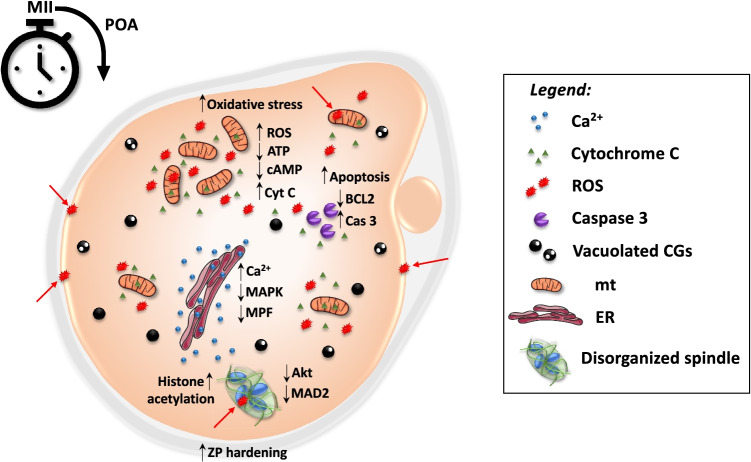
Main POA-related morphological and molecular alterations in mammalian oocytes. After ovulation of the MII-stage oocyte, POA-related mechanisms occur in unfertilized oocytes. Morphological alterations include ZP hardening, mt and CGs abnormal distribution patterns, CGs vacuolization, and spindle organization. On a molecular level, POA induces the increase of intracellular ROS levels and oxidative stress mechanisms, inducing lipids peroxidation, mt and DNA oxidative damages (arrows), and Ca^2+^ release from ER. Oxidative stress and cytochrome C release from mt activate apoptotic mechanisms that, together with higher intracellular Ca^2+^ levels, inactivate the cell cycle regulators (MPF, MAPK) and destabilize the epigenetic pattern (i.e., histone acetylation). Abbreviations: ATP, adenosine triphosphate; Ca^2+^, calcium; cAMP, cyclic adenosine monophosphate; Cas 3, caspase 3; CGs, cortical granules; Cyt C, cytochrome C; ER, endoplasmic reticulum; MAPK, mitogen-activated protein kinase, MII: metaphase II; MPF, maturation promoting factor; mt, mitochondria; POA, postovulatory aging; ROS, reactive oxygen species; ZP, zona pellucida

The current review will focus only on POA-related mechanisms (Fig. 1) that we kept separately from preovulatory aging. The latter involves the female age-related damage of oocytes that cumulates along the advanced maternal age. Although the biological consequences of post and preovulatory oocyte aging can largely overlap and will elevate the risks for fertilization failure, compromised embryo viability, miscarriage, and offspring diseases, the molecular mechanism of both types of oocyte aging can be uniquely distinguished.

## A brief overview of *in vivo* and *in vitro* oocyte meiotic maturation

Since POA can occur either *in vivo* or *in vitro*, the time-dependent degradation/inactivation of proteins involved in nuclear and cytoplasmic remodeling plays a key role in fertilization failure and disrupted embryo viability.

It is universally accepted that the production of a fertilizable oocyte requires the coordinate development of both germ and somatic granulosa cells (GCs) from primordial up to preovulatory stage [5]. In terminally differentiated follicle, the fully grown oocyte, still arrested at diplotene of prophase I (germinal vesicle, GV), is closely surrounded by several thousands of specialized GCs, the cumulus cells (CCs); the other GCs, the mural granulosa cells, line the follicular wall and define the fluid-filled antral cavity [4, 5]. The main feature of oocyte meiotic maturation is the achievement of an MII-arrested haploid oocyte, and this process is regulated by similar molecules in animals as in humans [13].

Oocytes are arrested at GV stage until the luteinizing hormone (LH) surge. This arrest is maintained by adequate levels of cyclic adenosine monophosphate (cAMP) that are finely regulated by a balanced mechanism of synthesis and degradation [6]. Surrounding GCs transmit to the oocyte signals involved in the regulation of intraoocyte cAMP levels. This regulation is based on a regulative loop [14], stimulated not only by follicle stimulating hormone (FSH) but also by sex hormones, as androgen and estrogen [15, 16].

The midcycle surge of LH determines several irreversible effects [17], including activation of the epidermal growth factor (EGF), EGF/EGFR pathway [18]. EGFR signaling in turn regulates CCs expansion and coordinates oocyte cytoplasmic maturation, thereby influencing the developmental capacity of the oocyte [19, 20].

The activation of the maturation promoting factor (MPF) triggers the germinal vesicle breakdown (GVBD) and chromosome segregation via securin degradation [21, 22]. Following GVBD, meiosis I is completed by discharging the PBI with a haploid set of chromosomes; subsequently, the oocytes enter meiosis II but arrest at MII stage until fertilization because of a high MPF activity [23]. This resting state can be stable for many hours and is dependent on a cytoplasmic activity termed CSF, which inhibits the anaphase-promoting complex/cyclosome (APC/C). Once the oocyte will be fertilized, APC/C will become active and cyclin B degraded. Members of the Emi/Erp family of proteins are components of CSF: Emi2, which is controlled by Mos-mitogen-activated protein kinase (MAPK) pathway, competitively inhibits APC/C binding before fertilization, while its degradation after fertilization reduces its inhibitory action [24, 25].

Along with nuclear maturation, cytoplasmic maturation of oocytes implies the synthesis, activation, and degradation of maternal mRNA, as well the rearrangement of organelles, especially CGs, mitochondria (mt), endoplasmic reticulum (ER), and cytoskeleton (for more details: [26]). Usually, a low oocyte quality correlates with abnormal mt and ER rearrangement. In fact, alterations of adenosine triphosphate (ATP) production of free Ca^2+^ storage/release in the oocyte cytoplasm as well as of microtubules/microfilament dynamics compromise irreversibly fertilization process [27].

*In vitro* maturation (IVM) is a technology by which immature oocytes retrieved from antral follicles can be maintained in a suitable culture medium until reaching the MII stage. This definition however is not sufficient for human IVM, since the stimulation protocols, follicle size, and time of oocyte retrieval influence its outcome. In humans, IVM is considered restricted to a limited number of patients such as those with polycystic ovary syndrome, history of ovarian hyperstimulation syndrome or poor responders. During ART procedures, some GV and metaphase I (MI) oocytes can be retrieved from antral follicles. The finding that the *in vitro* culture of immature oocytes seems to improve more nuclear than cytoplasmic maturation [28] suggests that these events can occur simultaneously but independently of each other. Although the comparison of *in vivo* and *in vitro* matured human oocytes reveals many morphological similarities [29–31], extending culture period can lead to POA and increased risk of aneuploidies.

## Postovulatory aging *in vivo* studies

Delayed fertilization of ovulated oocytes leads to the activation of a plethora of biochemical and molecular mechanisms that impairs oocyte quality and jeopardizes the success of embryo formation and development [32].

In order to better understand the mechanisms underlying this phenomenon *in vivo*, studies have been conducted using animal models, particularly mouse and rat, by retrieving the ovulated oocytes after several hours’ post-human chorionic gonadotropin (hCG) injection. In this section, we report the main findings present in literature.

### Morphological alterations

During the past years, several *in vivo* studies demonstrated interesting findings on the effect of POA on the fine oocyte structure, which manifest as a variety of morphological alterations that impact reproductive outcome. Postovulatory changes occur in a short time. CGs distribution is altered, because some areas appeared to be free of CGs, which are in part vacuolated, in part internalized into the ooplasm, and in part swollen [33]. Numerous dense structures, vesicles (lysosome-like vesicles) are present, and ooplasmic fragmentation is often observed. This could be related to the apoptotic phenomenon described in aged mouse oocytes [33]. Similar changes have been found in mouse oocytes 24 h after ovulation: the percentage of swollen CGs that are internalized from the membrane into the central region of the oocyte increases, as they lose their anchorage in the cortex [34, 35]. In mice, *in vivo* administration of caffeine, a potent antioxidant, causes, on the one hand, a significant decrease in the normal oocyte percentage, and, on the other hand, premature CGs exocytosis in low-quality oocytes, and a delay in exocytosis and congregation of CGs in good-quality oocytes [36]. In addition, the decreased numbers of CGs, together with a time-dependent increase in CGs exocytosis, as well as zona pellucida (ZP) hardening, have been observed in mouse POA-oocytes [37–39]. *In vivo* short-term resveratrol treatment has no impact on mt distribution, nor in preventing spindle aberrations in mouse POA-oocytes. In fact, Liang and coworkers found that microtubules displayed two distribution patterns, a homogeneous and a clustered one, in both resveratrol-treated and control groups. Also, they detected the presence of a variety of altered spindles as abnormal, elongated, without poles, mono-polar, multi-polar, and unorganized spindles with astral microtubules, together with the presence of many ooplasmic asters [40]. Similarly, Wortzman and Evans described microtubule aster-like bundles in the ooplasm and in the cortex of POA-oocytes in mice [41]. A 25-h-long aging causes altered structure of ER and mt network, facilitating formation of the cistern aggregates [42]. Also, chromosomal abnormalities have been reported in unfertilized *in vivo* POA-oocytes [43], and the presence of distended amicrovillar domains has been evidenced by after 21–22-h post-hCG [44]. Interestingly, Dalo and collaborators in mouse POA oocytes described three different phenotypes of microvillar and amicrovillar domains that are all equally correlated with the impaired ability of being fertilized [45]. In particular, the first phenotype shows a normal microvillar surface, increased fertilization cones with wrinkled appearance; the second shows a microvillar region where occasionally are present in bulb-like vesicles and small distinct pits, vesicles related to the precocious exocytosis and CGs loss [37]; the third one is with small amicrovillar patches over the site of sperm incorporation.

### Oxidative stress, ATP, and mitochondrial membrane potential

cAMP is universally recognized as the key regulator of GV oocytes meiotic maturation arrest. Nonetheless, the cyclic nucleotide, ATP, and AMP-activated protein kinase (AMPK) are involved in MII-stage oocyte arrest before fertilization [9, 46]. After ovulation, the MII-stage oocyte is released in the fallopian tubes, thus being subjected to different environmental stimuli, as the oxidative stress (OxS) that, if prolonged in time, activate several biochemical and molecular processes intended for egg activation [47, 48].

Igarashi and collaborators investigated modifications in intracellular ATP concentration in fresh and *in vivo* mouse POA-oocytes [49]. The authors suggested that aged oocytes exposed to increased level of OxS undergo a dysregulation of mt ATP production that produces an incomplete readjustment of ATP levels at fertilization. ATP dysregulation could directly or indirectly (via altered Ca^2+^ oscillations) affect the oocyte quality and, after fertilization, the embryo development [49]. To further confirm mt role in POA-oocytes, the same research group investigated mt membrane potential (MMP), oxygen consumption rate and mitochondrial transcriptional factor A (TFAM) [50]. Results showed that in POA-oocytes, both MMP and oxygen consumption rates were significantly lower compared to fresh oocytes. Taken together, the mt instability results in incomplete readjustment of ATP levels, leading to an impaired intracellular Ca^2+^ homeostasis and to a poor embryonic development [50]. On the other hand, while the total TFAM expression was similar between fresh and POA-oocytes, its colocalization with mt decreased with time, suggesting an impaired mt biogenesis. It is noteworthy that attempts to rescue oocyte fertilization quality by microinjecting mt from other somatic cell types into aged oocytes failed in both mice [50] and women [51].

The previous results are further confirmed by a recent study from Sun and collaborators that reported a significant decrease (almost 40%) in cAMP levels in mouse oocytes collected from the fallopian tubes after 24 h from ovulation [46]. The decline in cAMP, followed by inactivation of protein kinase A, promoted AMPK activation that facilitated oocyte aging through the inhibition of MPF downstream signaling via increasing ROS.

These data are connected directly and indirectly to the presence or the release of ROS in POA-oocytes, where a gradual accumulation of hydrogen peroxide, super-oxide anion, and peroxynitrite reactive compounds occurs [3, 11]. Sirtuin (SIRT) family genes are highly relevant to OxS, since their modulation is correlated with ROS presence, and the expression of these genes helps the recovery of a more stabilized cellular environment [52–54]. Zhang and collaborators demonstrated that oocyte mRNA levels of *Sirt1*, *2*, and *3* significantly decreased in a time-dependent manner [55]. In mice, literature data corroborate that short-term injections of resveratrol, a natural phenolic compound/phytoalexin, effectively ameliorated OxS-induced damage by increasing the mRNA and protein expression of SIRT1, reducing the ROS intracellular levels and improving mt function [40]. If not stabilized by the antioxidant machinery of the cell, the increasing level of ROS in the cytoplasm often results in damaged cellular and mt proteins, lipids, and DNA, one of the major OxS targets [3].

### Intracellular Ca^2+^ homeostasis

As mentioned above, OxS is responsible for the activation of several molecules and factors that subsequently alter downstream biochemical and molecular mechanisms. To this, both OxS and ATP/cAMP dysregulation result in impaired Ca^2+^ homeostasis [52], linked to ER stress-related environment [56].

In rat, Premkumar and Chaube demonstrated that an insufficient increase in free intracellular Ca^2+^ induces POA-derived abortive spontaneous egg activation (SEA) in a time-dependent fashion (~ 60% after 17-h post-hCG injection, and 100% after 19 h) [8]. The importance of intracellular Ca^2+^ level is corroborated by recent studies that found abnormal Ca^2+^ oscillations in mouse POA-oocytes compared to fresh ovulated oocytes [57, 58], correlating it to higher fragmentation and altered mt membrane permeability which leads to the release of proapoptotic factors [57]. Specifically, the source of Ca^2+^-impaired homeostasis is linked to ER-specific ATPase activity suppression, depletion of Ca^2+^ levels in ER [59], and inositol 1,4,5-trisphosphate (IP_3_)-dependent mechanisms misfunction [60]. These processes are due to ER stress environment, as demonstrated by Takehara and collaborators that noticed a significant increase in *Grp78*, an ER stress-related marker [56].

Overall, the unbalanced Ca^2+^ levels caused by a prolonged retaining period in the oviducts may interfere with later processes crucial for sperm fertilization in mice [61] and with parthenogenetic activation and male pronuclei formation after ICSI in golden hamsters [62].

### Cell cycle regulatory factors and spindle-associated proteins

One of the mechanisms affected by delayed fertilization is cell cycle regulation, mainly via the downregulation of MPF and MAPK proteins [63]. As direct or indirect consequence of both cAMP and Ca^2+^ altered levels, MPF inactivation induces oocyte activation. On the one hand, cAMP-dependent AMPK activation gradually inactivates MPF in a timely manner (from 13 to 24 h after hCG injection) [46]. On the other hand, higher levels of Ca^2+^ activates calmodulin-dependent protein kinases that destabilize MPF through the APC/C activation of MPF regulator, Wee1. This kinase triggers the dissociation of cyclin B1 from cyclin-dependent kinase 1 (Cdk1), leading to its ubiquitination and degradation and PBII extrusion [9, 46].

Mimicking MPF trend, MAPK1/3 reduced activity is demonstrated in both mouse [39] and cat oocytes [64]. In *in vivo* mouse POA-oocytes, kinase levels undergo a significant decrease, affecting the phosphorylation of the myosin-II regulatory light chain and increasing the rates of parthenogenetic activation [44].

As MPF and MAPK, the spindle-associated kinase Akt1/2/3 is highly deregulated during POA, together with the translocation of γ-tubulin from spindle poles to microtubules [65, 66]. Specifically, the mRNAs of all the isoforms are drastically reduced during in vivo POA, and the phosphorylated forms (Ser473- and Thr308-pAkt), generally associated with spindle poles disappear during POA in a time-dependent fashion [65]. Moreover, the absence of actin-rich cap or the presence of an abnormal actin-rich protrusion over the meiotic spindle has been observed in mouse POA oocytes after 24–48-h post-hCG [41]. Finally, also the reduced expression of *Mad2* transcript found after 19- and 24-h post-hCG can contribute to abnormal spindle functionality in the process of chromosomal segregation [67], together with the translationally controlled tumor protein, which has been shown to be necessary for spindle assembly dynamics in oocytes during POA [68]. Taken together, these results suggest a propension of the POA-oocyte to meiotic errors and unfavorable conditions for either fertilization or successful embryo development.

### Apoptosis

DNA fragmentation, mt dysfunction, and impaired Ca^2+^ homeostasis caused by high OxS often result in the release of pro-apoptotic factors (e.g., cytochrome c), and the dysregulation of anti-apoptotic factors (e.g., BCL2) has been frequently observed in *in vivo* POA-oocytes [11, 42, 69]. Transcriptional control of anti- and pro-apoptotic markers has been also linked to the differential expression of apoptosis-related microRNAs (miRNAs) during in vivo POA [70]. In their study, Wang and collaborators highlighted the presence of 6 upregulated miRNAs related to apoptosis, of which one promotes anti-apoptotic mechanisms and 5 promote pro-apoptotic mechanisms, as the upregulation of caspase 3 through miR-98 [70]. The increased activity of caspase 3 leads to increased intracellular Ca^2+^ levels and, subsequently, increased STAS levels. Moreover, the increment in miR-15a and miR-16 copies could be related to a post-transcriptional regulation of BCL2 [71].

## Postovulatory aging *in vitro* studies

**Fig. 2:**
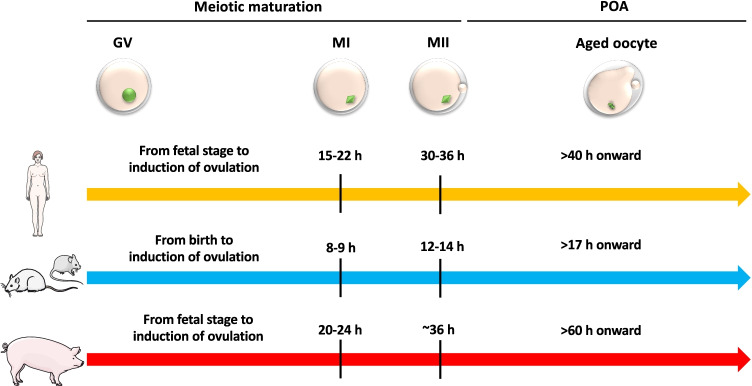
Timeline of meiotic maturation and POA in *in vitro* studies in different mammals: human, mouse, rat, and pig. Schematic summary of the average different timings utilized for meiotic maturation and activation of POA in the *in vitro* studies reported in this review. For humans, see [13]. Abbreviations: GV, germinal vesicle; MI, metaphase I; MII, metaphase II; POA, postovulatory aging

To deepen the knowledge in POA-inducing mechanisms and to test the effectiveness of “antiaging” molecules, *in vitro* studies have been conducted in humans and animal models, among which rat, mouse, and pig, by retrieving the ovulated MII-stage oocytes and culturing them in the presence or absence of antioxidant molecules and other natural/chemical additive to modulate POA (Table 1). Timings of *in vitro* experimental studies included in this review are summarized in Fig. 2. In this section, we report the main findings present in literature that suggest the use of this “antiaging” molecules as a potential tool against in vitro POA during ART procedures.

**Table 1:** Antiaging chemicals beneficial effects in treated in vitro POA-oocytes

Antiaging chemical	Beneficial effects	Species	References
NAC	↑ Spindle organization ↑ CGs and mt distribution ↓ ROS ↑ ATP	Mouse	[72–74]
Melatonin	↑ CGs distribution ↓ Cytoplasm fragmentation ↑ Cytoskeleton organization ↓ ROS ↑ *GPX4*, *POLG2* ↑ MMP ↑ BCL2 ↓ Bax, Bad	Mouse Pig	[75] [76]
Resveratrol	↑ Spindle morphology ↑ Chromosome alignment ↓ ROS ↑ BCL2 ↓ Caspase 3	Mouse Pig	[77, 78] [79]
CoQ10	↑ Spindle organization ↑ Chromosome alignment ↑ CGs and mt distribution ↓ ROS	Mouse	[80]
Astaxanthin	↑ Spindle organization ↑ mt, ER, Golgi apparatus, and lysosomes functions ↓ ROS	Pig	[81]
Ubiquinol-10	↑ Cytoskeleton organization ↑ Spindle organization ↓ ROS ↑ ATP ↑ *SIRT1*, *PGC1A* ↑ *ATG5, ATG7* and *LAMP2*	Pig	[82]
Quercetin	↑ Spindle organization ↑ Chromosome alignment ↑ mt distribution ↓ ROS ↑ *Sirt1*, *Sirt2*, *Sirt3* ↓ Phosphorylated-Cdk1 ↑ MAPK1/3, cyclin B ↑ BCL2 ↓ Caspase 3	Mouse	[83]
Icariin	↑ Spindle organization ↑ Chromosome alignment ↑ mt distribution ↓ ROS	Pig	[46]
*Artemisia asiatica*	↑ Spindle organization ↑ Chromosome alignment ↑ mt distribution ↓ ROS ↑ BCL2	Mouse	[84]
Bezafibrate	↓ ROS ↑ GSH ↓ Caspase 3	Pig	[85]
Imperatorin	↓ ROS ↑ MMP ↑ GSH ↑ SOD, CAT ↑ BCL2 ↑ *ATG5*, *ATG7*, and *LAMP2*	Pig	[86]
Caffein	↑ SIRTs ↓ Ca^2+^	Mouse	[63, 87]
Putrescine	↓ ROS ↑ SOD2, SIRT1, FOXO3 ↑ MMP ↓ Phosphorylated-Cdk1 ↑ MAPK1/3, cyclin B ↑ BCL2 ↓ Caspase 3 and 9	Pig	[60]
RO-3306	↓ Phosphorylated-Cdk1 ↑ Cyclin B1	Rat	[88]

Abbreviations: *ATG*, autophagy protein; *Bad*, BCL2-associated agonist of cell death; *Bax*, BCL2-associated X protein; *BCL2*, B-cell lymphoma 2; *CAT*, catalase; *Cdk1*, cyclin-dependent kinase 1; *CGs*, cortical granules; *CoQ10*, coenzyme Q10; *FOXO3*, forkhead box protein O3; *GPX4*. phospholipid hydroperoxide glutathione peroxidase; *GSH*, glutathione; *LAMP2*, lysosome-associated membrane glycoprotein 2; *MAPK*, mitogen-activated protein kinase; *MMP*, mitochondrial membrane potential; *mt*, mitochondria; *NAC*, N-acetyl-L-cysteine; *PGC1A*, peroxisome proliferator-activated receptor gamma coactivator 1-α; *POLG2*, mitochondrial DNA polymerase subunit gamma-2; *SOD*, superoxide dismutase

### Morphological alterations

*In vitro* POA seems to have a greater impact on oocyte morphology and many studies focus on the caused organelle alterations of oocytes that may limit the reproductive outcome. In mice, *in vitro* POA-oocytes surrounded by CCs showed a partial CGs exocytosis and ZP hardening, while very few CC-free oocytes released their CGs, suggesting that CCs may have a role in fastening POA processes *in vitro* [39]. Additionally, caffeine supplementation caused an acceleration of the abnormal CGs distribution and an increase of CGs exocytosis [36], in accordance with the *in vivo* findings reported above. Morphological observations in mouse aged oocytes revealed aggregates of ER cisterns and of mt [42], similar to those reported *in vivo*, and an increase in the abundance of large autophagic lysosomes and in spindle length [89]. After 24 and 48 h, a defective spindle assembly and a loss of mt normal distribution — completely or partially — and of normal CGs localization in the subcortex have been detected in porcine oocytes [90]. Also in *in vitro* mouse POA-oocytes, increased frequency of spindle defects and abnormal distribution of mt pattern have been found after treatment with nicotinamide (NAM), indicating that inhibition of sirtuin family members (SIRT1, 2, 3) may accelerate degenerative processes [55]. Mouse oocytes cultured for 12 h, 18 h, and 24 h showed increased percentages of abnormal spindles, mt distribution, and CGs distribution, and the treatment with N-acetyl-L-cysteine (NAC) antioxidant helped in alleviating these modifications [79]. Similar findings regarding the improvement of abnormal CGs distribution have been described when melatonin was added *in vitro* during oocyte culture [91]. However, even if resveratrol treatment had a beneficial effect on spindle integrity, morphology, and chromosomal alignment in *in vitro* mouse POA-oocytes, as well as on mt distribution, the CGs distribution was still found to be disturbed [92, 93]. Coenzyme Q10 (CoQ10) supplementation in mouse POA-oocytes aged for 24 h *in vitro* recovered spindle assembly, misalignment of chromosome, abnormal distribution patterns of mt, and CGs distribution (discontinued or completely disappeared signals of CGs) [94]. Jia and collaborators also found abnormal spindles, as condensation, elongation, dispersal, or disruption occurred and chromosome failed to align at the metaphase plate in *in vitro* porcine POA-oocytes [95], but astaxanthin supplement showed to maintain spindle organization and the functional status of mt, ER, Golgi apparatus, and lysosomes. Ubiquinol-10 rescued aging-induced cytoskeleton impairment and even if POA caused an increase in abnormal spindles, ubiquinol-10 decreased the rate of abnormal spindles at both 24 and 48 h of aging, in pigs [96]. Oocyte morphological defect and increased mt aggregation have been found in mouse oocytes aged 24 h *in vitro* [72]. However, dose-dependent melatonin treatments partially restored these morphological defects [72]. Oocytes aged for 24 h *in vitro* mostly showed normal morphology with low fragmentation, while after 48 h, their ooplasm was non-uniform and it was dispersed being dark pigmented [75]. More than half of the 48-h porcine POA-oocytes became fragmented, but melatonin treatment improved the abnormal morphology and contributed to decrease fragmentation percentage [75]. Disruption or loss of the microfilament domain underlying the plasma membrane was found in *in vitro* porcine POA-oocytes [77]. Notably, a study on human POA-oocytes showed altered ultrastructure of ZP and of organelles, as evidenced by a reduction of mt-smooth ER aggregates size and amount, an increase of mt-vesicle complexes size and amount, a decrease of CGs and microvilli, and alterations of the spindle structure [78]. However, quercetin, icariin, and *Artemisia asiatica* treatments attenuated the aging-associated morphological abnormalities [80–82]. Notably, Ogawa and coworkers demonstrated an improvement of the developmental competence of *in vitro* POA-oocytes through the MII spindle transfer in mice [97].

### Oxidative stress, ATP, and mitochondrial membrane potential

*In vitro* studies in different animal models showed an increase in ROS production during POA, as demonstrated in porcine [75, 76, 83, 90, 95, 96, 98, 99] and murine species [46, 55, 72, 79–81, 84, 91, 93, 100]. In mice, supplementation of NAC antioxidant used at 0.6 mM showed a decrease in ROS production and further OxS-related mechanisms during *in vitro* POA after 18–24 h, followed by an increase of ATP levels [79, 85]. Also in porcine oocytes, Niu and collaborators evidenced the protective effects of ubiquinol-10 against ROS increment and ATP/mt activity depletion [96]. Furthermore, the authors found an improved mt biogenesis, through the measurement of mRNA expression of mt genes as *SIRT1* and *PGC1A* in ubiquinol-10-treated POA-oocytes [96].

Melatonin treatment *in vitro* appears to relieve in a time-dependent manner the initial OxS process activation [1]. In mouse [91] and porcine [75] oocytes, literature data demonstrated that melatonin-supplemented culture helps in significantly decreasing ROS levels, along with increasing the transcription of anti-OxS gene *GPX4* and mt-related gene *POLG2*, and the recovery in MMP. On the contrary, melatonin treatments are not enough for increasing intracellular glutathione levels nor the transcription of anti-oxidative and mt related genes, as *SOD1*, *CAT*, *CYCS*, *SIRT1*, and *AKT2* [75]. Similar results are obtained when oocytes are cultured in the presence of resveratrol (mouse [93], pig [76]), extracts of *Artemisia asiatica* [81], astaxanthin [95], bezafibrate (Bez) [98], CoQ10 [94], imperatorin [101], and icariin [82].

The different levels in cytoplasmic ROS may also be connected to the protected role of SIRT proteins, as demonstrated in mouse POA-oocytes treated with the SIRT1/2/3 inhibitor, NAM. After 6 and 12 h of exposure to 5 mM NAM, a SIRT-dependent increase in ROS levels is detected in comparison with control oocytes [55]. On the other hand, treatment with caffein 10 mM delayed the POA process by stimulating the transcription of SIRTs [55]. The important role of SIRTs has been proved also in mouse POA-oocytes treated with quercetin [80], that showed a dose-dependent preventive action in *Sirt-1*, -*2*, and -*3* transcriptional levels decrease, leading to reduced ROS accumulation and spindle abnormalities. Similarly, in porcine POA-oocytes treated with putrescine, Xu and collaborators reported intracellular ROS decrease, and SOD2, SIRT1, and FOXO3 mRNA and protein expression level rescue, together with an increased MMP index [83]. Notably, the dysfunction provoked by an abnormal MMP index, and its connection to ROS, AMPK,and cAMP levels, has been proved in mouse POA-oocytes treated with metformin [46]. In brief, metformin induces the production of ROS after the activation of AMPK, leading to a decline in cAMP intracellular levels, destabilizing the MMP and facilitating POA mechanisms [46]. In addition, the imperatorin-supplemented culture of porcine oocytes enhance the MMP, oxidation resistance through enzymatic activation of SOD and CAT, and GSH levels in porcine POA-oocytes [101].

### Intracellular Ca^2+^ homeostasis

As POA-related mechanisms can induce abortive SEA, Premkumar and Chaube demonstrated the importance of intracellular Ca^2+^ balance and oscillations by culturing rat oocytes in the presence or absence of the Ca^2+^ channel blocker nifedipine. The results show how a partial increase in Ca^2+^ levels (promoted by nifedipine) can induce abortive SEA, while a further increase (induced by Ca^2+^ supplementation) can induce physiological egg activation [8]. In both mouse and pig oocytes, literature data link the alteration in Ca^2+^ oscillation to its depletion from ER stores and, subsequently, release of proapoptotic factors triggered by increased MMP, resulting in POA-oocytes fragmentation [42, 99]. POA-related ER stress and dysfunction induce biochemical modification of IP_3_R1, thus impairing the correct release of Ca^2+^ from ER that can be prevented by caffeine *in vitro* treatment [60].

Notably, using the Kinex™ KAM-850 Antibody Microarray for the detection of protein kinases, phosphatases, and related activators/regulators, McGinnis and collaborators found that Ca^2+^/calmodulin-dependent protein kinases (CAMKs) family members were overexpressed (> 30%) in mouse POA-oocytes after just 8 h of culture [89]. This result, together with the decreased expression of Ca^2+^ binding proteins, calnexin, and calreticulin, shows an impairment in Ca^2+^ homeostasis [89]. Moreover, Sun and collaborators found that the inhibition of CAMKs through STO-609 together with oocyte metformin-treatment increased levels of active AMPK during POA processes, suggesting a link between AMPK, Ca^2+^ signaling and CAMKs [46].

### Cell cycle regulatory factors and spindle-associated proteins

Alike with *in vivo* POA studies, when MII-ovulated oocytes are cultured *in vitro*, the cell cycle factors are subjected to deregulation, thus accelerating degenerative mechanisms. In rat, mouse, and porcine oocytes, *in vitro* POA increased Cdk1 Thr14/Tyr15 phosphorylation and activity and decreased cyclin B1 levels, thus destabilizing MPF activity [46, 80, 102, 103]. The use of RO-3306, a Cdk1 inhibitor, prevents Cdk1 phosphorylation and further cyclin B1 ubiquitination, providing stability for MPF and delaying POA [102]. The deregulation of MPF is generally featured by an early MAPK inactivation [83, 103, 104] and cell cycle-related kinases and phosphatases, as Chek1 and 2, Cdc25c [89]. These degenerating processes can be delayed by supplementing culture medium with antioxidants (e.g., quercetin) and polyamines (e.g., putrescine) that act by downregulating Cdk1 Tyr15 phosphorylation and MAPK1/3 inhibition, and upregulating cyclin B [80, 83].

Interestingly, McGinnis and coworkers found an extensive deregulation of spindle-associated proteins and kinases involved in spindle assembly and stability, as SRC-family proteins, ABL, and the aurora kinase A, B, and C [89]. In mouse oocytes, *in vitro* POA mechanisms are responsible for time-dependent downregulation of MAD2 [10, 104], and the formation of the actin cap over the spindle [91], causing destabilization in the spindle formation and errors in sister chromatin segregation. The supplementation of melatonin in cultured oocytes can reduce the rate of aberration in actin filaments formation and spindle-related abnormalities [91].

### Apoptosis

After the first hours of *in vitro* oocyte culture (both murine and porcine), the occurrence of POA-related OxS coincides with the activation of early apoptosis mechanisms, including phosphatidylserine externalization and BCL2 downregulation [1, 76, 80, 81, 89, 90, 93, 103, 104]. The activation of cell death mechanisms can be prevented by a treatment of *in vitro* POA-oocytes with melatonin [1] that inhibits the decrease of expression of BCL2 and the overexpression of pro-apoptotic genes Bax and Bad [80], as well as with putrescine [83], extracts of *Artemisia asiatica* [81], quercetin [80], resveratrol [76, 93], and imperatorin [101].

The use of antiaging molecules resulted often also in a decreased detection of caspases in POA-oocytes, suggesting a protective role of these supplements during oocyte culture. Particularly, caspase 3 downregulation has been proved in the presence of resveratrol [76], quercetin [80], and Bez [98]. Putrescine-treated oocytes display a recovery in caspase 3 and caspase 9 activation that were significantly reduced after 24 h of culture in porcine oocytes [83].

Notably, recent literature data demonstrated the activation also of autophagy markers in *in vitro* POA-oocytes [73]. In particular, the supplementation with ubiquitinol-10 and imperatorin appeared to prevent the overexpression of apoptosis- and autophagy-related genes, as *CASP3*, *SURVIVIN*, *ATG5*, *ATG7*, and *LAMP2*, after 24 h of culture [96, 101].

## Epigenomic and genomic aberrations in postovulatory aging

In the last decades, various groups have evidenced that POA mechanisms can interfere with the normal epigenetic asset before and after fertilization, thus affecting embryo epigenome and its further development [74, 86]. *In vivo* and *in vitro* studies examined DNA methylation among the epigenetic modifications and found a POA-dependent loss of methylation in the CpG of maternal imprinting genes *Snrpn* and *Peg1/Mest* after 13 h of culture and 29-h post-hCG injection [87, 88]. Furthermore, studies in mouse POA-oocytes detected a significant aging-dependent increase in pericentromeric ATP-dependent helicase (ATRX) [105], together with abundance in histone H4K12 acetylation and pericentromeric histone H3K9 demethylation [93, 105]. These findings are in accordance with studies in mice and pigs reporting increased histone acetylation also in H3K14 and H4K8 [64, 65, 106–108]. On the contrary, Huang and coworkers showed an accelerated aging process when mouse oocytes treated for 5 h with trichostatin A, due to raising levels of histone acetylation [106].

A study from Dankert and collaborators investigated transcript levels and their posttranscriptional poly(A) tail length of maternal effect genes [109]. The quantitative RT-PCR analysis revealed a significant shortening in poly(A) tails of *Nlrp5*, *Tet3*, *Trim28*, and *Dnmt1*, after both in vivo and in vitro POA [110]. These results, together with the chromatin and histone modification, suggest that POA may induce modification in the epigenetics of aging oocytes, leading to poor fertilization rates and embryo development.

Even more profound effect of POA has been found on genomic constitution of mammalian oocytes. Due to the scarcity of human oocytes, animal models have been mostly used to accumulate the evidence on the importance of POA on decreased oocyte quality and compromised viability of *in vitro* produced embryos generated from POA-oocytes. As POA studies focus on the phenomenon occurring with the ovulated oocytes, the POA concerns mostly MII oocytes that have successfully completed the first meiotic division separating the paired homologous chromosomes into MII oocyte and PBI, thus halving the chromosomal count from diploidy to haploidy. In MII oocytes, the chromosomes are made of the two sister chromatids that are aligned on the MII metaphase plate. The sister chromatids that are connected by adhesion factor, referred as cohesin, are oriented toward opposite spindle poles in a bipolar fashion by kinetochore protein structure mediating the connection between the centromeric DNA and meiotic spindle microtubules. Spindle assembly checkpoint (SAC), which operates during MII divisions, is one of the cell cycle checkpoint mechanisms that ensures that chromosomes are correctly attached and aligned to the spindle and are subsequently evenly segregated between the daughter cells. Protein MAD2 must be targeted to sister chromatids kinetochores, in order to accelerate the attachment of chromosomes to the spindle microtubules that ensures the proper meiotic segregation of chromosomes. It has been shown that in murine POA-oocytes, MAD2 localization to the sister kinetochores is inhibited, thus also the chromosome spindle attachment may remain incomplete [10]. In addition, POA prevents cohesin from being maintained or degraded at the appropriate time, thus destabilizing the SAC signaling and causing sister chromatid segregation errors, consequently leading to the higher incidence of aneuploidy in early embryos.

The mechanisms of POA in human oocytes are significantly more difficult to study, because of ethical restrictions, as human mature oocytes cannot be used for research, at least not in sufficient quantities. However, the IVF procedure, as the most commonly practiced ART procedure, provides a good opportunity to better understand the mechanisms of POA. IVF clinics today are large medical facilities where procedures can be delayed due to the excessive workload of clinical and laboratory staff. In addition, there may be a situation where a semen sample arrives at the laboratory significantly later than expected. All of this can cause the delay between the oocyte retrieval and the planned timing of insemination or ICSI. The timing of performing the injection of oocytes after denudation has been shown to be a critical factor determining the pregnancy success of ICSI. Indeed, recently, the analysis of more than 3600 ICSI treatments revealed that a long time from denudation to ICSI was associated with significantly decreased clinical pregnancy rate when compared to the cycles with short interval of up to 4 h [111]. Although this study did not reveal any possible POA mechanisms, the results suggest that POA in oocytes, especially in artificial environment, begins fairly rapidly after oocyte retrieval and must be considered more carefully in clinical practice. In addition, the POA process may be accelerated by the fact that oocyte culture conditions in the IVF/ICSI procedure do not yet meet ideal physiological conditions and may therefore be one of the reasons for the acceleration of POA.

A more direct evidence of POA’s effect on the deteriorated oocyte quality has been provided by IVF procedures with unexpected fertilization failure. In these unfortunate cases, IVF is done on retrieved mature oocytes but without fertilization, while rescue ICSI is done after 24 h using *in vitro* aged oocytes. The pregnancy results following “rescue ICSI”-derived embryos have remained relatively modest, which likely indicates the deteriorating effect of *in vitro* POA on mature human oocytes. There is only a single report providing the information on chromosomal aberrations in these embryos disclosing the high rate of diverse chromosomal aberrations [112]. As a conclusion, the use of “rescue ICSI” embryos was discouraged due to the observed increase in chromosomal abnormalities in these embryos, likely manifesting the negative effect of POA on human embryos.

## Environmental chemicals and oocyte quality

As discussed above, multiple chemicals that naturally occur in the environment, such as caffeine, resveratrol, and quercetin, have significant positive effects on oocyte quality *in vitro* and *in vivo*. Unfortunately, the environment also contains thousands of man-made chemicals [113], and some of them can disrupt the endocrine system being called endocrine-disrupting chemicals (EDCs) [114, 115]. Bisphenols and phthalates are examples of EDCs that are used in plastics and cosmetics, and perfluoroalkyl substances are surface-active chemicals used in food packaging, firefighting foam and non-sticky textiles, and kitchen utensils. Some man-made chemicals, such as chlorinated pesticides (e.g., DDT) and polychlorinated biphenyls (PCBs), have been restricted internationally due to their toxic properties, but they still widely contaminate food stock due to their extreme persistent. Ubiquitous exposure to pesticides, PCBs, phenols, fluoroalkyl compounds, flame retardants, and perchlorate can be found in Americans including pregnant mothers [116, 117]. Exposure starts in the womb, as EDCs can be found in umbilical cord blood and fetal tissues [110, 118, 119]. EDCs can also be detected in human ovarian follicular fluid; PCBs, pesticides, and PFAS are present virtually in all ART patients studied, and the levels in serum and follicular fluid are roughly the same [120]. High levels of PCBs in serum of women correlate with lower ovarian reserve, poorer embryo quality, and longer time-to-pregnancy, suggesting that chemical exposures can accelerate reproductive aging [121, 122]. Collectively, these results imply that chemical contaminants may affect oocyte quality, especially by impairing mitochondrial activity [123–125], thereby lessening the chances for fertilization. There are no studies using *in vitro* exposure of human oocytes to EDCs to investigate impact on maturation and POA, but animal studies strongly support the sensitivity of mammalian oocytes toward low-level EDC exposure. For example, exposure of bovine cumulus-oocyte-complexes during IVM to PFOS (a perfluoroalkyl substance used in firefighting foam) at levels found in human ovarian follicular fluid leads to a delayed cleavage development, altered lipid distribution, and epigenetic and gene expression changes that persist to blastocyst stage [126]. Similarly, exposure of mice to bisphenol A via damaged polycarbonate cages or oral exposure leads to congression failure in oocytes [127]. A realistic scenario is that the developmental competence of oocytes exposed for years to these chemicals can be dramatically impaired *per se*, and that it could be further worsened by the degenerative processes induced by POA, either *in vivo* or *in vitro*. Significant research remains to be accomplished, including studies of EDCs that may leak from plastics used during IVF treatments, to fully understand the consequences of man-made chemicals on POA.

## Conclusions

Literature data clearly evidence that POA causes a number of morphological, molecular, genomic, and epigenomic aberrations in oocytes and related embryos, the mechanisms of which are beginning to be gradually acknowledged. However, it can be assumed that POA plays a major role in determining the impaired environment, by increasing ROS levels and oxidative stress, capable of affecting developmental potential of oocytes and embryos, pivotal in IVF/ICSI cycles. As genetic and epigenetic alterations may be passed on to the next generation and cause health issues and diseases, the mechanisms of *in vitro* POA should be investigated in much more detail in the future. The present review strongly suggests that avoiding POA and the use of “antiaging” molecules during *in vitro* culture may be key-elements to improve the overall performance of the IVF procedure.
